# Safety and efficacy of AST-001 in children with autism spectrum disorder: a 52-week multicenter long-term follow-up study

**DOI:** 10.3389/fpsyt.2026.1783915

**Published:** 2026-06-01

**Authors:** Johanna Inhyang Kim, Hyo-Won Kim, Ji-Hoon Kim, MoonSoo Lee, Su-Kyeong Hwang, Yoo-Sook Joung

**Affiliations:** 1 Department of Psychiatry, Hanyang University College of Medicine, Seoul, Republic of Korea; 2 Department of Psychiatry, University of Ulsan College of Medicine, Asan Medical Center, Seoul, Republic of Korea; 3 Department of Psychiatry, Pusan National University Yangsan Hospital, Yangsan, Republic of Korea; 4 Department of Psychiatry, Korea University, College of Medicine, Seoul, Republic of Korea; 5 Department of Pediatrics, School of Medicine, Kyungpook National University, Daegu, Republic of Korea; 6 Astrogen Inc., Daegu, Republic of Korea; 7 Department of Psychiatry, Sungkyunkwan University School of Medicine, Samsung Medical Center, Seoul, Republic of Korea

**Keywords:** AST-001, autism, children, clinical trial, long term safety

## Abstract

**Background:**

This study examined the 52-week safety and efficacy of AST-001 syrup for the core symptoms of autism spectrum disorder (ASD) in children aged 2–11 years.

**Methods:**

This multicenter long-term follow-up study enrolled children with ASD who were identified as responders in an antecedent phase II randomized controlled trial. A total of 61 participants were given the same dose (high or low) they received during the extension phase of the phase-II trial for 52 weeks. Safety and tolerability were evaluated by reporting adverse events. Efficacy was assessed using the Ohio State University Autism Clinical Global Impression (CGI)-Severity and Improvement scale.

**Results:**

The proportion of participants experiencing any treatment-emergent adverse events was 40.98%, most commonly due to COVID-19 (11/61 participants, 18.03%), but none were related to AST-001. Two participants experienced adverse drug reactions (2/61 participants, 3.28%), including decreased appetite and enteritis. The incidence of serious adverse events was 4.92% (3/61 participants), which were dental caries, otitis media, and septic shock, but none were related to medication. The mean CGI-S score decreased slightly from 4.25 ± 0.87 at baseline to 4.10 ± 0.79 at week 52 of the long-term follow-up study. Among the 48 participants who were CGI-I responders at week 24 of phase-II trial, 45 continued to meet the responder criteria at week 52.

**Conclusions:**

AST-001 was well tolerated over the 52-week period, and clinical improvement was maintained among participants who had previously responded to treatment, suggesting continued benefit on ASD core symptoms. These findings provide preliminary long-term data on safety, tolerability, and clinical outcomes in a field where pharmacologic treatment options for the core symptoms of ASD remain limited. However, given the uncontrolled long-term follow-up design in prior responders, the results should be interpreted with caution and do not constitute confirmatory evidence of sustained efficacy. Further large-scale, well-controlled studies are warranted.

**Clinical Trial Registration:**

https://cris.nih.go.kr/cris/search/detailSearch.do?seq=26492&search_page=L&search_lang=&class_yn=, identifier KCT0007519.

## Introduction

Autism spectrum disorder (ASD) is a neurodevelopmental disorder that affects about 1% of children worldwide (1). Core symptoms of ASD include deficits in social interactions and restricted and repetitive behaviors and interests (2). The etiology of ASD is poorly understood, and no medication has been proved to alleviate its core symptoms (3). Novel medications, including cholinergic and glutamatergic agents, intranasal oxytocin (4), and vasopressin 1a receptor antagonists (5) have been investigated, but the study results failed to show substantial improvement in core ASD symptoms compared to the placebo. The current direction of treatment is to implement various multidisciplinary nonpharmacological interventions, including applied behavior analysis (ABA), language, and physical therapy (6).

Although the exact neurobiology underlying ASD is unclear, imbalance in dopamine levels in the mesolimbic dopamine pathway leads to impairment in social interactions—a core deficit in ASD (7). The mesolimbic pathway connects the ventral tegmental area (VTA) of the midbrain and the nucleus accumbens (NAc) of the striatum (8). The NAc and VTA regions respond to social stimuli and regulates social motivations (9), as endorsed by fMRI studies reporting reduced NAc activation in children and adults with ASD (10). Another prominent hypothesis is the excitatory/inhibitory imbalance hypothesis, which suggests that an imbalance between the excitatory (predominantly glutamatergic) and inhibitory (predominantly GABAergic) mechanisms underlies ASD symptomatology (11).

Recent studies have reported AST-001, an L-isomer of serine, as a promising therapeutic agent for the core symptoms of ASD. AST-001 acts as modulators of the small conductance Ca^2+^-activated K^+^ channel (SK channel) in dopamine neurons (12). Administration of AST-001 improved sociability and social novelty by rescuing the intrinsic excitabilities of dopamine neurons in valproic acid-exposed ASD mouse models that showed ASD-related behavioral abnormalities (13). AST-001 also modulates the excitatory/inhibitory neurotransmission pathway in prefrontal cortex neurons related to the regulation of core ASD symptoms (12).

In a previous randomized, double-blind, placebo-controlled phase-II trial of AST-001 (14), the mean Vineland Adaptive Behavior Scales, 2^nd^ Edition (VABS-II) Adaptive Behavior Composite (ABC) score at 12 weeks significantly improved in the high-dose AST-001 group compared to the control group. Moreover, the mean Clinical Global Impression-Severity (CGI-S) score significantly decreased in both the high-dose and low-dose AST-001 groups compared to the control group, providing preliminary evidence for AST-001 in improving the core ASD symptoms. AST-001 was also safe as no significant difference in the incidence of adverse events (AEs) related to the medication was found between the treatment and placebo groups. However, the long-term efficacy and safety of this agent have not been determined in patients with ASD.

This study was conducted to examine the long-term (52-week) safety and efficacy of AST-001 syrup on the core ASD symptoms in a pediatric population with ASD. We also compared the safety and efficacy of AST-001 in high-dose and low-dose groups.

## Methods

This was a 52-week multicenter long-term follow-up study that followed an antecedent phase II 36-week double-blind randomized placebo-controlled trial (14). It consisted of low-dose and high-dose arms, and both the participants and raters were blind to group status. The phase-II trial was conducted from February 2021 to May 2022, while this long-term follow-up study was conducted from January 2022 to May 2023. Children and adolescents with ASD were recruited from 5 of the 10 hospitals that participated in the phase-II trial in South Korea.

### Participants

All participants had to meet the following inclusion criteria: (i) age between 2 and 11 years at the time of participation in the phase-II trial; (ii) had a diagnosis of ASD defined by the Diagnostic and Statistical Manual of Mental Disorders, Fifth Edition, Text Revision (DSM-5) and corroborated by the Autism Diagnostic Interview-Revised (ADI-R); (iii) enrolled in the phase-II trial (Protocol No. AST-001P_P201_ASD) and completed the end-of-study visit (EOS, 36 weeks); (iv) defined as responders who had a therapeutic response to AST-001 in the phase-II trial by having shown an increase in the K-VABS-II ABC score of 4 or more points from baseline to end of treatment (EOT) at 24 weeks or received a very much improved (1 point) or much improved (2 points) CGI-I score at week 24 (EOT).

Participants were excluded from the study according to the following exclusion criteria: (i) schizophrenia, other psychosis, or mood disorders, including bipolar disorder and major depression according to the DSM-5 criteria; (ii) organic brain disease, neurological disorder, uncontrolled epilepsy, or a history of seizures, except for simple febrile seizures; (iii) multisystem genetic disorder; (iv) sensory abnormalities such as congenital hearing loss; (v) severe self-harm or injury to others that requires medical treatment; (vi) digestive disorder or surgical history that may influence intestinal absorption of AST-001; (vii) history of allergy to serine injections or serine supplements; and (viii) weight exceeding 60 kg at the time of enrollment in the phase-II trial. During the study period, the use of any concomitant medications that could affect the efficacy or safety of the investigational product (IP) was prohibited. This included.psychotropic medication classes (e.g., antipsychotics such as risperidone and aripiprazole, psychostimulants, antidepressants, anxiolytics, mood stabilizers or neuroleptics, and anticonvulsants), as well as medications reported or investigated to influence ASD-related symptoms (e.g., neuropeptides or hormones such as oxytocin and secretin, NMDA or glutamatergic modulators such as memantine, acamprosate, and riluzole, cholinesterase inhibitors such as rivastigmine and donepezil, loop diuretics such as bumetanide, folate derivatives such as folinic acid, growth factor therapy such as mecasermin, vitamin D3, vasopressin receptor antagonists, and nicotinic receptor agonists). In this follow-up study, indication-related non-pharmacological therapy was not separately collected, and it was guided that pre-existing non-pharmacological therapy in the Phase-II study be maintained.

### Study design

**Figure 1:**
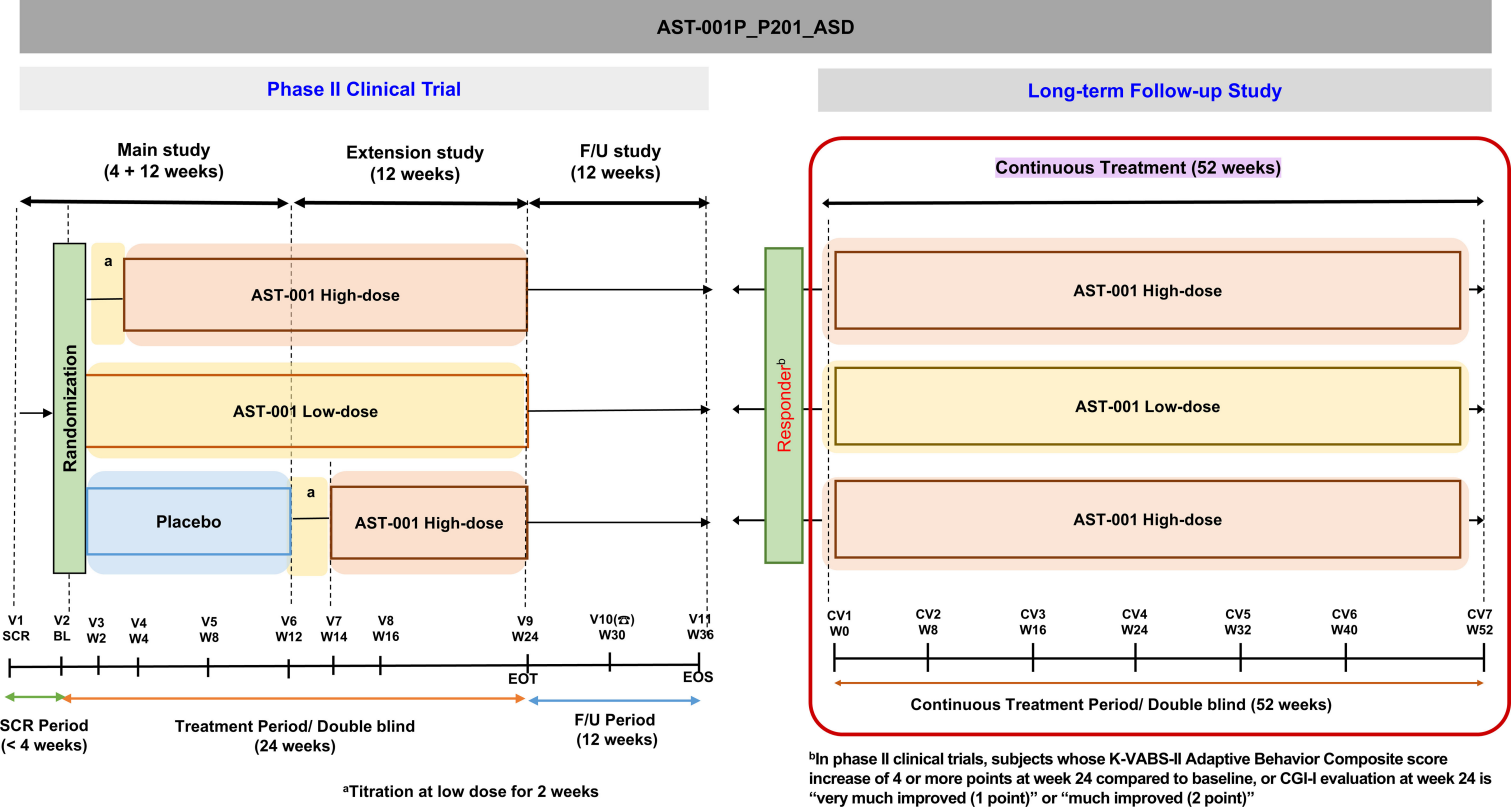
Study design of a phase II clinical trial and long-term follow-up study. CGI-I, clinical global impression-improvement; F/U, follow up; K-VABS-II, Korean-vineland adaptive behavior scales, second edition; SCR, screening.

This trial is a 52-week long-term follow-up study subsequent to a 36-week phase-II clinical trial, which consisted of a 12-week main study (14), a 12-week extension study, and a 12-week follow-up study (**Figure 1**). All participants were treated according to a predefined body weight-tiered AST-001 regimen derived from model-informed dose selection. The supporting population PK model was developed using plasma concentration data from healthy adult subjects and incorporated body weight through allometric scaling of clearance and volume parameters. Model-based simulations supported fixed twice-daily regimens across five pediatric body weight ranges (10–14 kg, 15–24 kg, 25–37 kg, 38–51 kg, 52–60 kg), which were selected to provide adequate systemic exposure to AST-001 across the pediatric population (15). During the 12-week main study of the phase-II trial, participants were randomly assigned in a 1:1:1 ratio to the high-dose AST-001, low-dose AST-001, or placebo group. During the subsequent 12-week extension study, participants originally assigned to the high-dose and low-dose groups continued their assigned dose, whereas participants originally assigned to placebo were switched to high-dose AST-001. The end-of-study visit of the antecedent phase-II trial, which followed the 12-week follow-up period, served as the baseline for the present long-term follow-up study. In this 52-week long-term follow-up study, participants received AST-001 at the same dose level they had been receiving at the end of the extension phase of the phase-II trial. Accordingly, only low-dose and high-dose groups were carried forward into the long-term follow-up study, and no concurrent placebo group was included.

Throughout the entire study period, antipsychotics and medication that may influence the symptoms of ASD and natural/herbal medicine were restricted from use. Non-pharmacologic therapy for ASD (special education and other psychosocial treatment) was allowed and maintained throughout the study.

Comorbid psychiatric disorders, including attention-deficit hyperactivity disorder (ADHD), were included and diagnosed based on the DSM-5 criteria. Intellectual disability was based on standardized developmental assessments or intelligence tests.

### Assessment

We performed safety and efficacy assessments at baseline and at weeks 8, 16, 24, 32, 40, and 52. The baseline for the long-term follow-up study was defined as the end-of-study (EOS) visit of the phase-II trial. Each participant’s informant and rater remained consistent throughout the study.

At each visit, AEs (Adverse Events) were collected through open-ended questions directed at parents/guardians and/or patients, where feasible. A treatment-emergent adverse event (TEAE) was defined as any unfavorable medical occurrence in a patient receiving an investigational product (IP) that did not necessarily have a causal relationship with this drug. An adverse drug reaction (ADR) is any unfavorable and unintended AE occurring at any dose of IP, where causal relationship to IP cannot be ruled out. Accordingly, the causal relationship between AEs and the IP was evaluated by the investigator based on multiple factors, including the participant’s medical history, baseline conditions, and the temporal relationship to IP administration. Causality was categorized as related (certain, probable, possible, or not assessable) or not related (unlikely or not related). A serious adverse event (SAE) or serious adverse drug reaction (SADR) is AE or ADR occurring at any dose of IP that results in death, is life-threatening, requires inpatient hospitalization or prolongation of existing hospitalization, or results in persistent or significant disability/incapacity, congenital anomaly/birth defect, or other important medical events.

The efficacy measurement was the Ohio State University Autism Clinical Global Impression (CGI)-Severity and/or Improvement (16) score. The OSU Autism CGI includes two global ratings-Severity (CGI-S) and Improvement (CGI-I)-that provide an overall clinician-rated assessment integrating social communication and interaction impairments, restricted and repetitive behaviors, and clinically relevant associated behaviors. This clinician-rated global assessment adapted for ASD, integrating DSM-5 core symptoms and clinically relevant associated behaviors. The CGI-S reflects overall symptom burden at a given time point, while the CGI-I evaluates global change relative to baseline. In this study, the CGI-I score assessed change compared to the baseline of the phase-II trial. Rater training for the CGI was conducted both prior to the study and during the study as refresher sessions. These trainings included instruction on the general scoring principles of the CGI, as well as the evaluation of CGI ratings based on representative, non-study clinical case examples from multiple study sites. Through consensus discussions, standardized rating criteria were established, including an explicit consensus that symptoms referenced in the CGI reflected the core symptoms of ASD. In the long-term follow-up study, CGI-I responders were defined as participants with a CGI-I score of 1 (very much improved) or 2 (much improved) at each visit.

The long-term follow-up study was designed primarily to evaluate long-term safety and monitor clinical status over time; therefore, CGI-S and CGI-I were prespecified as the primary clinical assessments and were administered at each scheduled visit. K-VABS-II was not administered during the follow-up period to reduce participant/caregiver burden and the operational complexity associated with repeated caregiver interviews requiring trained raters.

### Statistical analysis

In the safety analysis set, we included all participants who received one or more doses of IP. For efficacy evaluation, we performed the main analysis on the full analysis set (FAS: intention-to-treat principle), which included those who received at least one dose of IP and had the CGI score, the primary efficacy endpoint, collected at baseline and at least once post-baseline time point. In the FAS analysis of efficacy endpoints missing data were imputed using the last observation carried forward (LOCF) method, whereby the most recent prior observed value was carried forward to replace missing value.

We compared demographic and clinical characteristics using two sample t-tests for continuous variables and Fisher’s exact tests for categorical variables.

For the TEAE, ADR, SAE, SADR, and AE that led to treatment discontinuation, the number of participants, incidence rate, and number of occurrences were presented by treatment group and analyzed by Fisher’s exact test. Additionally, the number of participants, incidence rate, and number of occurrences were coded by treatment group according to system organ class (SOC) and preferred term (PT) of the medical dictionary for regulatory activities (MedDRA).

For CGI-S results, we analyzed the significance of change from the baseline score of the long-term follow-up study to the score at each assessment period using paired t-tests. We analyzed comparisons between treatment groups (low-dose and high-dose) at each assessment point using two-sample t-tests or Wilcoxon rank sum tests depending on data normality. We used analysis of covariance (ANCOVA) to compare the mean changes in CGI-S score from the baseline of the long-term follow-up study to each time point between the treatment groups by including the baseline CGI-S score of the long-term follow-up study and age as covariates.

For CGI-I results, we analyzed comparisons between treatment groups at each time point using a two-sample t-test or Wilcoxon rank sum test depending on data normality.

For the difference between groups, the least squares mean difference, standard error (SE), 90% confidence interval (CI), and p-value of the change at each time point were presented. We performed all statistical analyses using SAS (version 9.4; SAS Institute Inc.) and set statistical significance at a two-sided 10% level of significance.

## Results

### Participants

**Figure 2:**
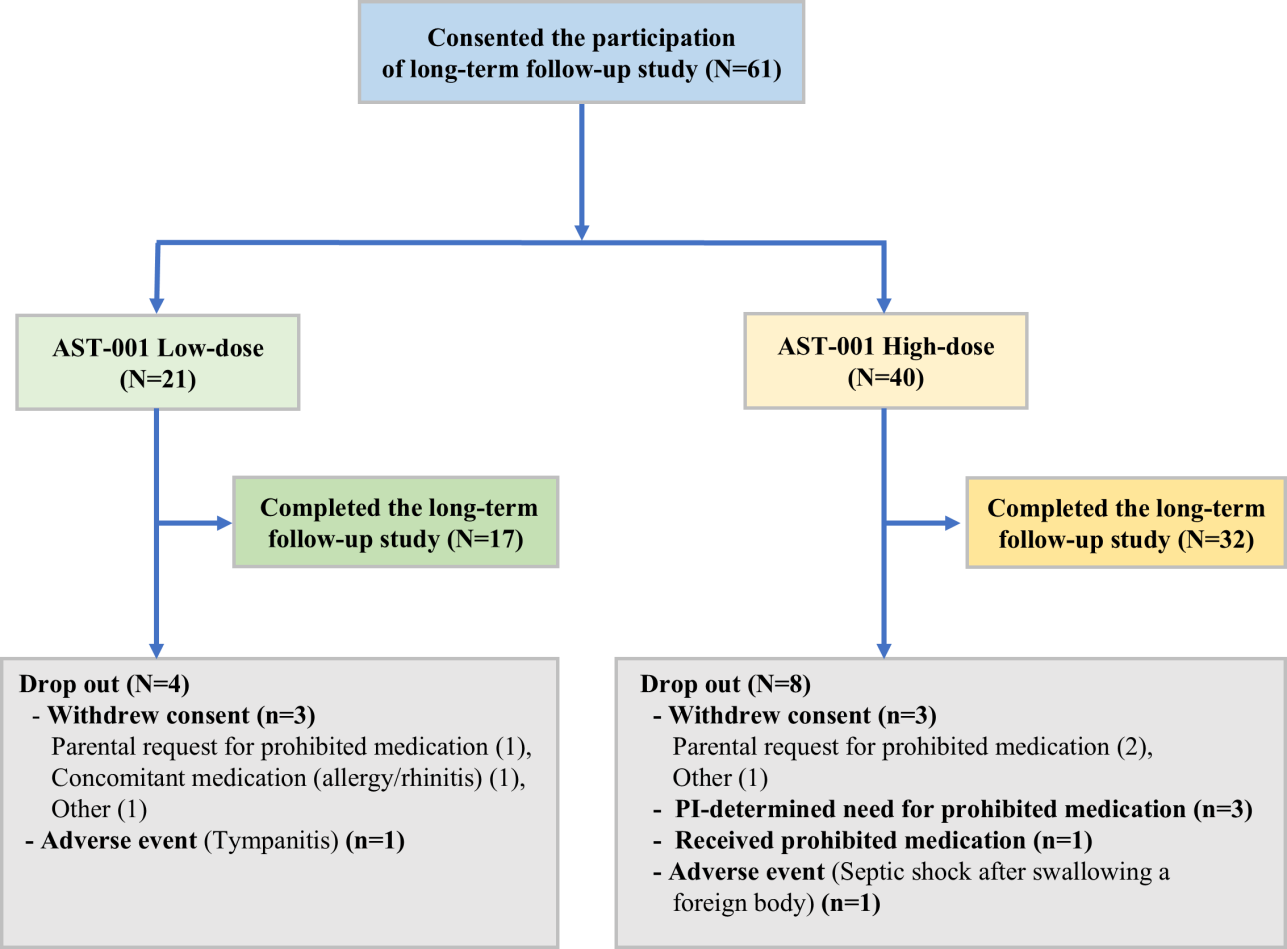
Participant disposition.

Among the 10 hospitals of the phase-II trial, five agreed to participate in the 52-week long-term follow-up study. A total of 102 participants from these five hospitals were enrolled in the phase-II trial, and 75 participants were identified as responders according to VABS-II or CGI-I score criteria. A total of 61 participants were enrolled in the 52-week long-term follow-up study, with 21 participants in the low-dose group and 40 in the high-dose group. This unequal distribution was attributable to the design of the antecedent phase-II trial rather than to re-randomization in the long-term follow-up study. In the 12-week main study of the phase-II trial, participants were initially randomized in a 1:1:1 ratio to the low-dose, high-dose, and control groups. During the subsequent 12-week extension study, participants originally assigned to the control group were switched to the high-dose regimen, whereas those in the low-dose and high-dose groups continued their assigned dose. In the present long-term follow-up study, participants received the same dose level that they had received at the end of the extension phase, resulting in a greater number of participants in the high-dose group. Forty-nine participants completed the 52-week trial (80.33%), with 17 in the low-dose group and 32 in the high-dose group. Among the 12 participants who dropped out of the study, 7 participants were related to prohibited concomitant medications because the PI determined that the use of prohibited concomitant medications was necessary, or because the parents withdrew consent to administer such medications. The participants’ disposition is displayed in **Figure 2**.

The participants’ demographic and clinical characteristics are shown in **Table 1**. The participants’ mean age was 6.23 ± 2.22 years, and most were male (n = 48, 78.69%). Fifty-two participants (85.25%) had a comorbid intellectual disability, and 13 participants (21.31%) had comorbid ADHD. The mean baseline CGI-S score of the long-term follow-up study was 4.25 ± 0.87. We found no statistically significant differences between the low-dose and high-dose group.

**Table 1:** Participants’ demographics and clinical characteristics.

Characteristics	Low-dose group	High-dose group	Total (N = 61)	p-value
	(N = 21)	(N = 40)		
Age^a^, years, mean ± SD	6.57 ± 2.64	6.05 ± 1.99	6.23 ± 2.22	0.388^c^
Sex, boys, n (%)	15 (71.43)	33 (82.50)	48 (78.69)	0.341^d^
Height^b^, cm, mean ± SD	121.24 ± 16.64	119.20 ± 14.25	119.90 ± 15.01	0.618^d^
Weight^b^, kg, mean ± SD	26.60 ± 11.91	26.13 ± 11.62	26.29 ± 11.62	0.881^d^
BMI^b^, kg/m^2^, mean ± SD	17.22 ± 2.77	17.58 ± 3.26	17.46 ± 3.08	0.666^d^
Comorbid psychiatry disorders				
Intellectual disability, n (%)	19 (90.48)	33 (82.50)	52 (85.25)	0.479^d^
ADHD, n (%)	4 (19.05)	9 (22.50)	13 (21.31)	1.000^d^
Baseline CGI-S score, mean ± SD				
Phase-II trial	4.86 ± 0.79	4.93 ± 0.89	4.90 ± 0.85	0.770^c^
Long-term follow-up study	4.29 ± 0.72	4.23 ± 0.95	4.25 ± 0.87	0.798^c^
Treatment duration, days, mean± SD	307.33 ± 106.23	317.43 ± 80.96	313.95 ± 89.69	0.680^c^

a age at time of participation in the long-term follow-up study.

b measured at Phase-II trial end of study visit (final visit).

c Two sample t-test.

d Fisher’s exact test.

ADHD, attention-deficit/hyperactivity disorder; CGI-S, Clinical Global Impression-Severity; SD, standard deviation.

### Safety outcomes

The summary of adverse events is shown in **Table 2**, and the types of TEAEs are presented in **Table 3**. The incidence of TEAEs during the entire study period was 40.98% (25/61 participants, 41 cases), with 57.14% (12/21 participants, 16 cases) in the low-dose group and 32.50% (13/40 participants, 25 cases) in the high-dose group. We found no significant difference in the incidence of TEAEs between the two groups (*p* = 0.100). Among 41 TEAEs, the most reported adverse event based on PT was “COVID-19” (18.03%, 11/61 participants, 11 cases). As this study was conducted during the COVID-19 pandemic, COVID-19 was the most common TEAE in both groups (five cases in the low-dose group and six cases in the high-dose group). When COVID-19–related events were excluded, the overall incidence of treatment-emergent adverse events was substantially lower, suggesting high tolerability of AST-001 during long-term administration. Most remaining TEAEs were reported in only one case (less than 5%) each. According to national public health guidance, COVID-19 diagnoses were confirmed by PCR testing and investigators collected COVID-19-related adverse event data based on confirmed test results.

**Table 2:** Summary of adverse events.

Safety outcomes	Low-dose group (N = 21)		High-dose group (N = 40)		Total (N = 61)		p-value^a^
	Number of participants (%)	Number of events	Number of participants (%)	Number of events	Number of participants (%)	Number of events	
TEAE	12 (57.14)	16	13 (32.50)	25	25 (40.98)	41	0.100
ADR	1 (4.76)	1	1 (2.50)	1	2 (3.28)	2	1.000
SAE	2 (9.52)	2	1 (2.50)	2	3 (4.92)	4	0.270
SADR	0	0	0	0	0	0	NA
AEs that led to discontinuing	3 (14.29)	4	3 (7.50)	7	6 (9.84)	11	0.406

a Fisher’s exact test.

Percentages were calculated using the number of participants in each group as the denominator. Several cases of adverse events can be collected from one patient.

TEAE, treatment-emergent adverse event; ADR, adverse drug reaction; SAE, serious adverse event; SADR, serious adverse drug reaction; NA, not applicable.

**Table 3:** Types of treatment-emergent adverse events (TEAEs).

TEAE	Low-dose group (N = 21)		High-dose group (N = 40)		Total (N = 61)	
	Number of participants (%)	Number of events	Number of participants (%)	Number of events	Number of participants (%)	Number of events
COVID-19	5 (23.81)	5	6 (15.00)	6	11 (18.03)	11
Cough	2 (9.52)	2	2 (5.00)	2	4 (6.56)	4
Pyrexia	0	0	3 (7.50)	3	3 (4.92)	3
Vomiting	1 (4.76)	1	1 (2.50)	2	2 (3.28)	3
Diarrhea	1 (4.76)	1	1 (2.50)	1	2 (3.28)	2
Decreased appetite	1 (4.76)^a^	1	1 (2.50)	1	2 (3.28)	2
Strabismus	1 (4.76)	1	0	0	1 (1.64)	1
Abdominal pain	0	0	1 (2.50)	1	1 (1.64)	1
Dental caries	1 (4.76)	1	0	0	1 (1.64)	1
Enteritis	0	0	1 (2. 50) ^†^	1	1 (1.64)	1
Food allergy	0	0	1 (2.50)	1	1 (1.64)	1
Hypersensitivity	0	0	1 (2.50)	1	1 (1.64)	1
Gastroenteritis bacterial	1 (4.76)	1	0	0	1 (1.64)	1
Laryngitis	0	0	1 (2.50)	1	1 (1.64)	1
Otitis media	1 (4.76)	1	0	0	1 (1.64)	1
Pneumonia	1 (4.76)	1	0	0	1 (1.64)	1
Septic shock	0	0	1 (2.50)	1	1 (1.64)	1
Foreign body ingestion	0	0	1 (2.50)	1	1 (1.64)	1
Benign neoplasm of skin	1 (4.76)	1	0	0	1 (1.64)	1
Aggression	0	0	1 (2.50)	1	1 (1.64)	1
Anger	0	0	1 (2.50)	1	1 (1.64)	1
Irritability	0	0	1 (2.50)	1	1 (1.64)	1
Total	12 (57.14)	16	13 (32.50)	25	25 (40.98)	41

a Adverse Drug Reaction.

Percentages were calculated using the number of participants in each group as the denominator. Several cases of adverse events can be collected from one patient.

MedDRA version 26.0.

The incidence of ADRs was 3.28% (2/61 participants, two cases), with 4.76% (1/21 participants, one case) in the low-dose group and 2.50% (1/40 participants, one case) in the high-dose group. The ADRs classified by PT were “decreased appetite” in the low-dose group and “enteritis” in the high-dose group. For both ADRs, the relationship with the IP was assessed as possible; severity was “Grade 1 (mild),” and the outcome was “recovered”.

The incidence of SAEs was 4.92% (3/61 participants, four cases), which was 9.52% (2/21 participants, two cases) in the low-dose group and 2.50% (1/40 participants, two cases) in the high-dose group. The SAEs classified by PT were dental caries and otitis media in the low-dose group and septic shock and foreign body ingestion in the high-dose group. For all SAEs, the relationship with the IP was reported as “not related”.

No SADR occurred, and the incidence of adverse events that led to treatment discontinuation was 9.84% (6/61 participants, 11 cases), which was 14.29% (3/21 participants, four cases) in the low-dose group and 7.50% (3/40 participants, seven cases) in the high-dose group. The adverse events leading to the treatment discontinuation, classified by PT were vomiting, otitis media, pneumonia, and decreased appetite in the low-dose group, and diarrhea, vomiting, pyrexia, COVID-19, aggression, irritability, and cough in the high-dose group. Among the adverse events that led to treatment discontinuation, 10 cases were unrelated to the IP. Only one case (decreased appetite in the low-dose group) was considered related, as the relationship with the IP was assessed as “possible.”

Among the 61 participants enrolled in this study, one 4-year-old male participant died of septic shock after foreign body ingestion and subsequent removal surgery, which was classified as a SAE. The severity of the SAE was grade 5 (death). Based on the death certificate, which identified septic shock due to peritonitis as the direct cause of death, the principal investigator (PI) determined that the SAE was not related to the IP and submitted an SAE report to the site Institutional Review Board (IRB). Following IRB review, the causal relationship of the adverse event was assessed as unrelated.

### Efficacy outcomes

**Figure 3:**
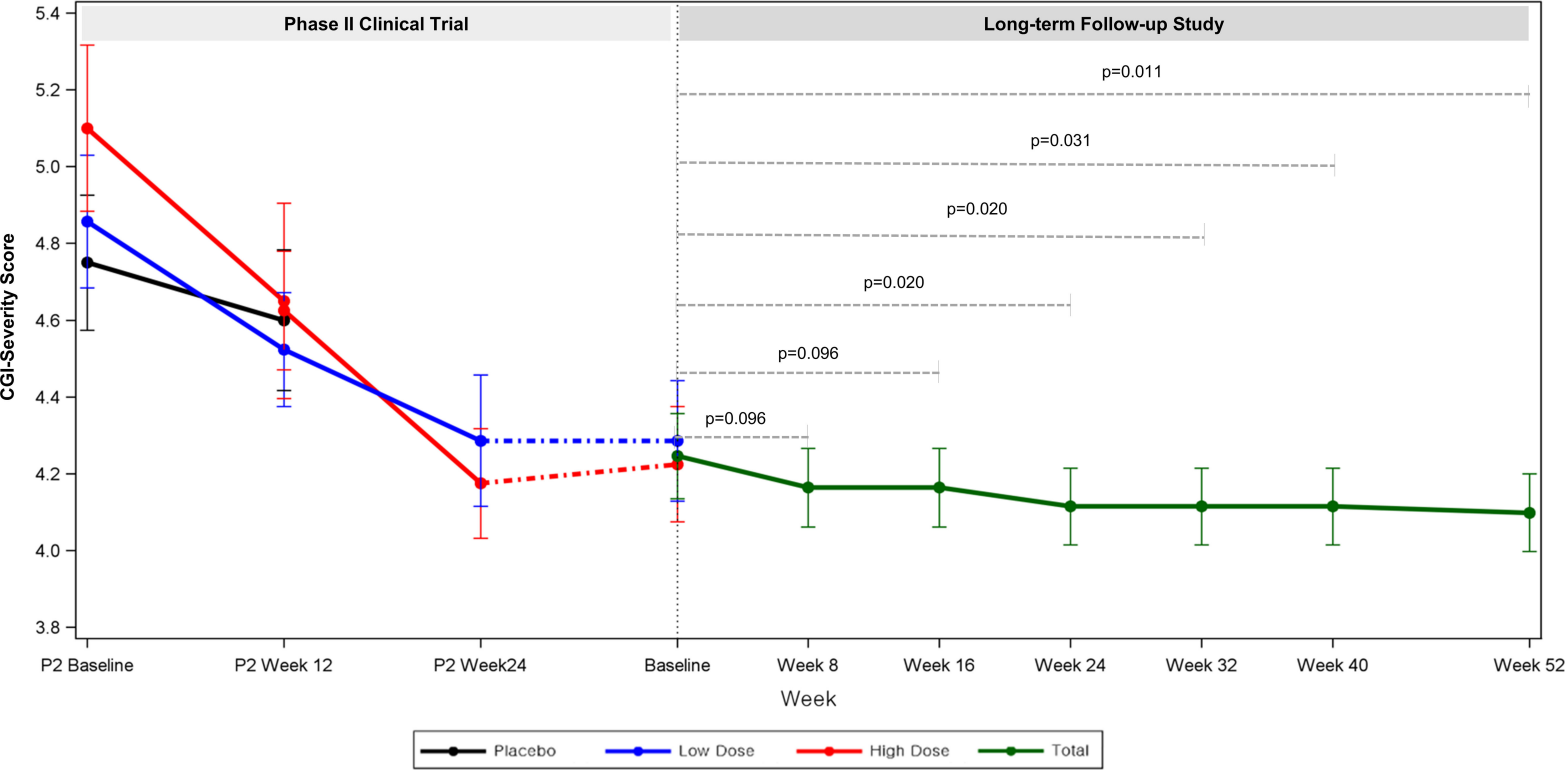
CGI-S scores over long-term follow-up compared to phase II baseline. CGI-S, clinical global impression-severity. The placebo group (black line) is shown through Phase II week 12 only; thereafter placebo group rolled over AST-001 high-dose group.

The mean CGI-S scores over 52 weeks are displayed in **Figure 3** and **Table 4**. The mean baseline CGI-S score of the long-term follow-up study was 4.25 ± 0.87, with 4.29 ± 0.72 in the low-dose group and 4.23 ± 0.95 in the high-dose group, which was numerically lower than the CGI-S score of the baseline of the phase-II trial (4.90 ± 0.85, with 4.86 ± 0.79 in the low-dose group ad 4.93 ± 0.89 in the high-dose group). The mean CGI-S score at week 52 was 4.10 ± 0.79, with 4.14 ± 0.65 in the low-dose group and 4.08 ± 0.86 in the high-dose group. Mean changes (mean ± SD) in CGI-S scores at each time point after administration were observed compared to the baseline of the long-term follow-up study. The changes in CGI-S scores were specifically -0.08 ± 0.38 points at week 8, -0.08 ± 0.38 points at week 16, -0.13 ± 0.43 points at week 24, -0.13 ± 0.43 points at week 32, -0.13 ± 0.46 points at week 40, and -0.15 ± 0.44 points at week 52 in the groups as a whole. P-values are presented for descriptive purposes only, and no adjustment for multiple comparisons was performed.

**Table 4:** Comparison of CGI-S scores by treatment group at each assessment period.

Treatment group	CGI-S score (mean ± SD)						
	Baseline	8 weeks	16 weeks	24 weeks	32 weeks	40 weeks	52 weeks
Low-dose (N = 21)	4.29 ± 0.72	4.19 ± 0.75	4.19 ± 0.75	4.14 ± 0.65	4.14 ± 0.65	4.14 ± 0.65	4.14 ± 0.65
High-dose (N = 40)	4.23 ± 0.95	4.15 ± 0.83	4.15 ± 0.83	4.10 ± 0.84	4.10 ± 0.84	4.10 ± 0.84	4.08 ± 0.86
p-value^a^	0.798	0.853	0.853	0.840	0.840	0.840	0.753
Total (N = 61)	4.25 ± 0.87	4.16 ± 0.80	4.16 ± 0.80	4.11 ± 0.78	4.11 ± 0.78	4.11 ± 0.78	4.10 ± 0.79

a Two sample t-test.

CGI-S, clinical global impression-severity; SD, standard deviation.

In the ANCOVA model adjusted for the baseline score of the 52-week long-term follow-up study and age, the difference (LS mean ± SE) in the change in CGI-S scores between the two groups at each time point after administration compared to the baseline of the long-term follow-up study was 0.01 ± 0.10 points at week 8 [90% CI: -0.15, 0.17], 0.01 ± 0.10 points at week 16 [90% CI: -0.15, 0.17], -0.00 ± 0.11 points at week 24 [90% CI: -0.18, 0.18], -0.00 ± 0.11 points at week 32 [90% CI: -0.18, 0.18], -0.01 ± 0.11 points at week 40 [90% CI: -0.20, 0.19], and -0.03 ± 0.11 points at week 52 [90% CI: -0.21, 0.15].

The CGI-I mean scores (mean ± SD) at each time point for the total group was 2.26 ± 0.54 points at baseline, 2.16 ± 0.49 points at week 8, 2.18 ± 0.53 points at week 16, 2.18 ± 0.56 points at week 24, 2.16 ± 0.52 points at week 32, 2.13 ± 0.53 points at week 40, and 2.13 ± 0.53 points at week 52 (**Table 5**). We found no statistically significant differences between the two groups at each time point.

**Table 5:** Comparison of CGI-I scores by treatment group at each assessment period.

Treatment group	CGI-I score (mean ± SD)						
	Baseline	8 weeks	16 weeks	24 weeks	32 weeks	40 weeks	52 weeks
Low-dose (N = 21)	2.19 ± 0.40	2.10 ± 0.44	2.14 ± 0.57	2.19 ± 0.60	2.14 ± 0.48	2.14 ± 0.48	2.14 ± 0.48
High-dose (N = 40)	2.30 ± 0.61	2.20 ± 0.52	2.20 ± 0.52	2.18 ± 0.55	2.18 ± 0.55	2.13 ± 0.56	2.13 ± 0.56
p-value^a^	0.460	0.431	0.694	0.920	0.822	0.902	0.902
Total (N = 61)	2.26 ± 0.54	2.16 ± 0.49	2.18 ± 0.53	2.18 ± 0.56	2.16 ± 0.52	2.13 ± 0.53	2.13 ± 0.53

a Two sample t-test.

CGI-I, clinical global impression-improvement; SD, standard deviation.

**Figure 4:**
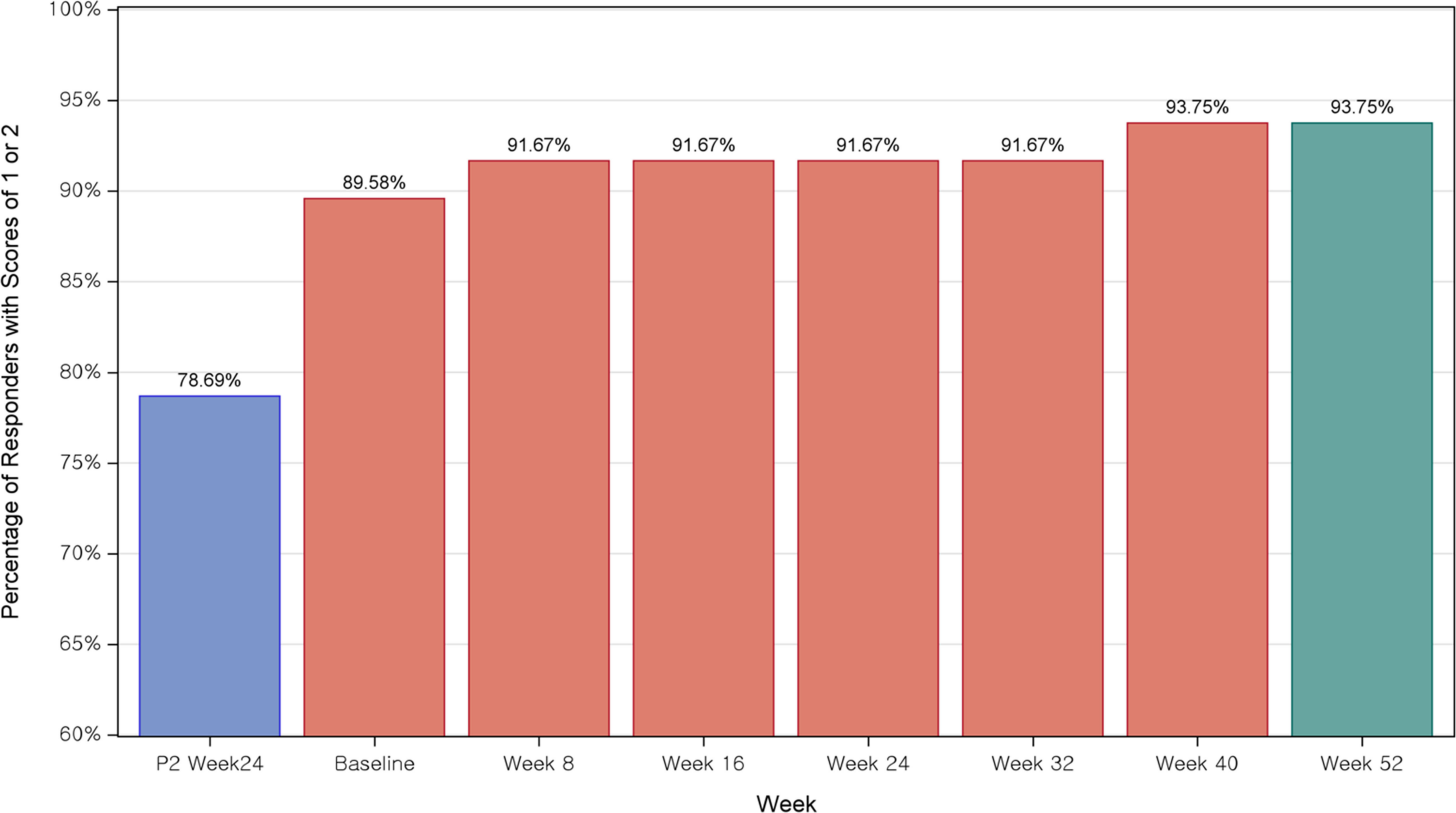
Proportion of CGI-I responders compared to EOT of phase II study.

Among the 61 participants, 48 (48/61 participants, 78.69%) were responders according to the CGI-I criteria at the end of the phase-II trial. Among these 48 participants, 43 (43/48 participants, 89.58%) were still responders at the baseline of the long-term follow-up study. The proportion of CGI-I sustained responders at each time point among all participants was 91.67% (44/48 participants) from 8 to 32 weeks, and 93.75% (45/48 participants) at 40 and 52 weeks (**Figure 4**). Throughout the study, more than 90% of participants continued to meet the CGI-I responder criteria during long-term follow-up period.

## Discussion

This study reported no serious adverse events related to AST-001, supporting the long-term safety and tolerability of AST-001 in children with ASD. CGI findings also suggested that overall clinical status was generally maintained over the 52-week long-term follow-up period, although interpretation of effectiveness remains limited by the uncontrolled design and potential confounding factors. Together with the antecedent phase-II trial, which included a protocol-defined 12-week follow-up period without study drug, the present study provides additional longitudinal safety and tolerability data beyond the double-blind treatment period.

Interpretation of between-group comparisons is limited by the design of the antecedent phase-II trial. The unequal group sizes in the present study arose because participants were initially randomized in a 1:1:1 ratio and control participants crossed over to the high-dose group during the extension phase, after which participants continued the same dose level in the long-term follow-up study. Although baseline characteristics were generally comparable, the relatively small sample size, unequal allocation, and absence of a concurrent control group reduced statistical power and limited the interpretability of dose-group differences. Accordingly, these between-group findings should be interpreted with caution.

Clinical research on L-serine has mostly targeted adults with various neurological and psychiatric disorders, including Parkinson’s disease (17), Alzheimer’s disease (18), epilepsy (19), schizophrenia (20), and amyotrophic lateral sclerosis (21). Recently, a 15-month, phase 2a, non-randomized study was conducted among patients with GRIN-related encephalopathy aged 2–18 years (22); L-serine was associated with improvement in adaptive behavior, motor function, and quality of life, and no serious adverse drug reactions were reported. Our 52-week study of children aged 2–11 years is in line with this previous study, which suggests that L-serine shows long-term tolerance in pediatric populations. Rather than providing confirmatory evidence of long-term efficacy, the main contribution of this study is to extend the limited evidence base on the longer-term tolerability and clinical follow-up of pharmacologic treatment for core ASD symptoms in young children.

Our study has several limitations. First, the overall sample size was relatively small, and the unequal group sizes (low-dose group: 21 participants, high-dose group: 40 participants) further limited statistical power for between-group comparisons. As noted above, this imbalance reflected the design of the antecedent phase-II trial rather than re-randomization in the long-term follow-up study. Although baseline characteristics were generally comparable, the imbalance may have reduced the interpretability of dose-group comparisons, and the absence of statistically significant differences should therefore be interpreted with caution. Second, our sample included only children aged 2–11 years, which limits the generalizability of our results to ASD in other age ranges. The safety and efficacy of AST-001 should be examined in other age groups. Furthermore, particularly in young children, changes in global clinical ratings over time may reflect not only treatment effects but also normative developmental maturation and expectancy-related (placebo) effects, which are known to be substantial in ASD clinical trials (23). Third, our results may differ in real-life clinical settings as our study was restricted by the exclusion of participants with concomitant medication use, medical and psychiatric comorbidities, and weight limitation. Fourth, despite its widespread use, CGI has several limitations. In long-term evaluations exceeding one year, CGI-I ratings relative to baseline may be particularly susceptible to rater subjectivity. Furthermore, as the CGI is not a norm-referenced measure, it has inherent limitations in accounting for age-related normative developmental effects. In addition, because the long-term follow-up study enrolled only responders from the phase-II trial and lacked a concurrent placebo comparator beyond the double-blind period, selection bias and expectancy/placebo effects may have contributed to the high observed responder rates, thereby limiting causal interpretation. Fifth, although psychosocial treatment was allowed if therapy could be maintained throughout the study, in some cases, minor changes in psychosocial treatment occurred due to the COVID-19 pandemic. In addition, because visit-level missing data occurred during the long-term follow-up study, LOCF method was used for summary analyses. However, LOCF may overestimate durability among participants who discontinue early, and the findings should therefore be interpreted with caution. Finally, our long-term follow-up study was limited to only responders of the previous phase-II trial.

Despite these limitations, AST-001 was well tolerated over 52 weeks and may contribute to maintaining overall clinical status in children with ASD. These findings provide supportive long-term follow-up data in a field where pharmacologic evidence for the core symptoms of ASD remains limited. Future studies should confirm these findings in larger, adequately controlled trials with broader ASD populations and outcome measures that better distinguish treatment-related change from developmental maturation and expectancy effects.

## Data Availability

The datasets presented in this article are not readily available because it contains information that could compromise the privacy of research participants. Requests to access the datasets should be directed to Yoo-Sook Joung, yschoung@skku.edu.

## Glossary

ABA: Applied Behavior Analysis
ABC: Adaptive Behavior Composite
ADHD: Attention-Deficit Hyperactivity Disorder
ADR: Adverse Drug Reaction
AE: Adverse Event
ANCOVA: analysis of Covariance
ASD: Autism Spectrum Disorder
CGI-I: Clinical Global Impression-Improvement
CGI-S: Clinical Global Impression-Severity
CI: Confidence Interval
DSM-5: Diagnostic and Statistical Manual of Mental Disorders, Fifth Edition, Text Revision
EOS: End-of-Study Visit
FAS: Full Analysis Set
GCP: Good Clinical Practice
IP: Investigational Product
LOCF: Last Observation Carried Forward
MedDRA: Medical Dictionary for Regulatory Activities
NAc: Nucleus Accumbens
PT: Preferred Term
SADR: Serious Adverse Drug Reaction
SAE: Serious Adverse Event
SD: Standard Deviation
SE: Standard Error
SK channel: Ca^2+^-activated K^+^ channel
SOC: System Organ Class
TEAE: Treatment-Emergent Adverse Event
VABS-II: Vineland Adaptive Behavior Scales, 2nd Edition
VTA: Ventral Tegmental Area
